# Evaluation of chronic radiation proctitis in patients with cervical cancer treated with pelvic radiotherapy: a cross-sectional study

**DOI:** 10.61622/rbgo/2025rbgo26

**Published:** 2025-05-04

**Authors:** Renata Silva Aragão, Candice Lima Santos, Ariani Impieri Souza, Ana Luiza Fassizoli da Fonte, Beatriz Ximenes Bandeira de Moraes, Lucas Sandes de Lima, Luiza Dias Aguiar, Jurema Telles Oliveira Lima

**Affiliations:** 1 Instituto de Medicina Integral Professor Fernando Figueira Recife PE Brazil Instituto de Medicina Integral Professor Fernando Figueira, Recife, PE, Brazil; 2 Faculdade Pernambucana de Saúde Recife PE Brazil Faculdade Pernambucana de Saúde, Recife, PE, Brazil

**Keywords:** Cervical neoplasms, Radiotherapy, Proctitis, Argon plasma coagulation

## Abstract

**Objective:**

A combination of chemotherapy and pelvic radiotherapy is recommended to treat locally advanced cervical cancer (CC), which has been associated with acute and chronic toxicities, especially radiation proctitis (RP). The objective of this study was to evaluate the frequency of RP and treatment management in females with CC who underwent pelvic radiotherapy at an oncology referral hospital.

**Methods:**

This cross-sectional study analyzed the medical records of patients treated with radiotherapy for CC between 2015–2017. We assessed sociodemographic, lifestyle, cancer, treatment, and clinical variables. We identified 298 records of females with CC who underwent pelvic radiotherapy during the defined period. Of these, 14 records were duplicates, 25 were excluded for lacking essential information, and 33 were missing in the archive. Accordingly, 226 relevant medical records were analyzed, with data regarding sociodemographic, clinical, cancer-related, treatment-related, and RP-related variables collected. Pearson's chi-square test was used to compare symptomatic and non-symptomatic patients. Fisher's exact test was used to compare chemotherapy doses. Statistical analysis was performed with Stata V12.1. A P-value less than 0.05 was considered significant.

**Results:**

The median patient age was 48 years (interquartile range 38–61). Patients predominantly had CC stages IIB and IIIB (>70%). Of the 226 females analyzed, 87(38.5%) experienced RP symptoms, represented by rectal bleeding; of these, 59 underwent colonoscopy, confirming RP in 58(98.3%). Accordingly, of the 226 females analyzed, 58(25.7%) had a confirmed diagnosis of RP. There was a statistically significant association between rectal bleeding and cumulative radiation dose (P < 0.001) and the presence of systemic arterial hypertension (P = 0.036). Regarding treatment, 38(65.5%) participants underwent argon plasma coagulation (APC), and of these, 22(57.9%) had no post-treatment macroscopic bleeding.

**Conclusion:**

Patients with CC who received radiotherapy at an oncology referral service had a high frequency of RP, and APC helped control bleeding in certain patients.

## Introduction

Cervical cancer (CC) is considered a public health challenge in Brazil, with 17,010 new cases estimated annually between 2023–2025 and a risk of 13.2 cases per 100,000 women.^(1)^

The initial clinical stages of CC include IA, IB1, and IB2, determined by the depth of stromal invasion and tumor size, and surgery is considered the most common treatment. More advanced stages (IB3 to IVA) present an increased risk of recurrence, and a combination of chemotherapy and pelvic radiotherapy is recommended.^(2)^ Pelvic radiotherapy is associated with gastrointestinal toxicity, commonly resulting in radiation proctitis (RP),^(3)^ which can affect any segment of the colon receiving treatment. However, the rectum is the most affected site owing to its fixed position in the pelvis.^(4)^

RP is defined as radiation-induced damage to the intestinal segment and can be differentiated into acute and chronic.^(5)^ Acute RP occurs during or within two to four weeks of radiotherapy initiation, with diarrhea documented as the most common symptom (50–75% of cases).^(6)^ Chronic RP is characterized by connective tissue fibrosis and obliterative endarteritis, with tissue ischemia and neovascular lesions in the mucosa.^(7)^ Chronic RP can occur from six months to several years after treatment initiation. The main symptom is bleeding^(8)^ caused by neoangiogenesis and vascular ectasia.^(9)^ Bleeding ranges from mild to moderate, resolving spontaneously or becoming chronic, associated with anemia and need for blood transfusions, negatively impacting the patient's quality of life.^(10)^

Given the lack of prospective studies, variable definitions and grading systems, the precise incidence of chronic RP remains unknown, and an incidence between 2–20% has been documented.^(11–14)^ RP onset is influenced by patient-related factors (age, hypertension, vasculopathies, diabetes, and smoking),^(15)^ as well as treatment-related factors (total radiation dose, fractionation type, technique used, number of fields used, and rectum volume).^(16,17)^ Endoscopy is employed as the main diagnostic tool, which can also establish the extent and severity of the disease.^(18)^

Diverse treatment options are available to combat chronic RP, from pharmacological to surgical and endoscopic interventions. Argon plasma coagulation (APC) is the most widely used endoscopic therapy, given its availability, portability, safety, and low cost. APC involves no contact with the mucosa and uses ionized argon gas at a high-frequency electric current for tissue coagulation.^(6)^ APC outcomes have been favorable, with complete or nearly complete resolution of rectal bleeding achieved in 80–90% of cases.^(19–22)^

Therefore, the objective of the current study was to determine the frequency of chronic RP and treatment management in patients with CC who received pelvic radiotherapy at an oncology referral hospital.

## Methods

This cross-sectional study assessed medical records of patients diagnosed with CC who underwent pelvic radiotherapy and/or brachytherapy at a teaching hospital that is part of the public health system in Recife, Brazil. We analyzed a census sample including all females treated with pelvic radiotherapy between January 2015 and December 2017.

We identified 298 records of females with CC who underwent pelvic radiotherapy during the defined period. Of these, 14 records were duplicates, 25 were excluded for lacking essential information, and 33 were missing in the archive. Accordingly, 226 relevant medical records were analyzed, with data regarding sociodemographic, clinical, cancer-related, treatment-related, and RP-related variables collected.

Pearson's chi-square test was used to compare symptomatic and non-symptomatic patients. Fisher's exact test was used to compare chemotherapy doses. Statistical analysis was performed with Stata V12.1. A P-value less than 0.05 was considered significant.

This study was approved by the Research Ethics Committee (Number:50034821.1.0000.5201) and no informed consent was required because this was a retrospective study based on existing data with no identifying details.

## Results

Of the 226 patients analyzed, 72, 65, and 89 underwent treatment in 2015, 2016, and 2017, respectively. The median patient age was 48 years (interquartile range [IQR] 38–61), and the mean age was 50.1 years (standard deviation [SD] 14.9). Evaluating lifestyle habits, 68(30.1%) patients were smokers. Regarding comorbidities, 22(9.7%) patients had diabetes mellitus (DM), 78(34.5%) had hypertension (HTN), and five had both diseases. Considering nutritional status there was a high frequency of normal weight, 70(30.9%) patients and overweight, 58(25.7%) patients (Table 1).

**Table 1:** Sociodemographic and clinical characteristics of females with cervical cancer who underwent pelvic radiotherapy

Characteristics		n(%)
Age (years)		
	Median: 48 (IQR* 38–61)	
	Mean: 50.1 years (SD^b^**14.9)	
	20–39	62(27.4)
	40–60	104(46.0)
	Above 60	60(26.6)
Hypertension		
	Yes	78(34.5)
	No	142(62.8)
	No information	6(2.7)
	Diabetes *Mellitus*	
	Yes	22(9.7)
	No	198(87.6)
	No information	6(2.7)
	Smoking	
	Yes	68(30.1)
	No	154(68.1)
	No information	4(1.77)
Nutritional status		
	Malnutrition	17(7.5)
	Normal weight	70(30.9)
	Overweight	58(25.7)
	Obesity	42(18.6)
	No information	39(17.3)

* IQR: interquartile range;

** SD: standard deviation

The predominant histological type was squamous cell carcinoma (86.7%), along with CC stages IIB (37.2%) and IIIB (36.7%). The primary treatments were radiotherapy with concomitant chemotherapy (84.1%) and radiotherapy alone (9.7%). We used 2D brachytherapy and the total dose was 28 Gy in four fractions of 7 Gy. This treatment was delivered sequentially to chemoradiotherapy. Brachytherapy was performed in 80.5% of patients (Table 2). Regarding radiotherapy, 193(85.4%) patients received a cumulative radiotherapy dose exceeding 8,500 cGy. Regarding the field, 202(89.4%) patients underwent radiotherapy in the pelvic field and 24(10.6%) in the pelvic field associated with the para-aortic field. Most patients underwent single-medication chemotherapy, particularly cisplatin associated with radiotherapy (68.1%). Thirty-three patients (14.6%) received cisplatin with gemcitabine, with 4.4% receiving protocol treatment including carboplatin.

**Table 2:** Tumor characteristics and treatment in females with cervical cancer undergoing radiotherapy

Characteristics		n(%)
Staging		
	IA	2(0.9)
	IB	15(6.6)
	IIA	7(3.1)
	IIB	84(37.2)
	IIIA	5(2.2)
	IIIB	83(36.7)
	IVA	19(8.4)
	IVB	9(4.0)
	No information	2(0.9)
Histology		
	Squamous cell carcinoma	196(86.7)
	Adenocarcinoma	24(10.7)
	Others	6(2.6)
Treatment		
	Radiotherapy + chemotherapy	190(84.1)
	Radiotherapy	22(9.7)
	Radiotherapy + Surgery + Chemotherapy	8(3.5)
	Radiotherapy + Surgery	6(2.7)
Brachytherapy		
	Yes	182(80.5)
	No	35(15.5)
	No information	9(4.0)

**Figure 1:**
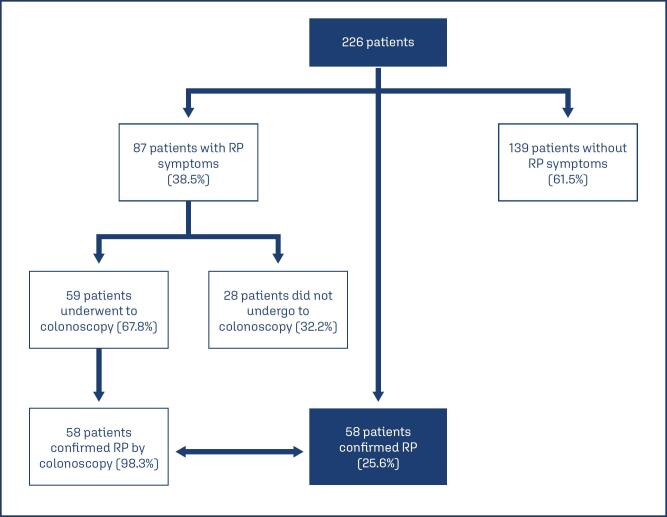
Radiation proctitis (RP) symptoms and diagnostic confirmation

Of the 226 patients, 87(38.5%) had RP symptoms, particularly rectal bleeding. Of the symptomatic patients, 59(67.8%) underwent colonoscopy to confirm RP, which was confirmed in 58(98.3%) patients. Thus, RP affected 25.6% of the patients (Figure 1).

Analyzing the time between the end of radiotherapy and RP onset, most cases occurred between six and twelve months (48.3%). The median time of follow up (defined as the time from diagnosis to last follow up) was 34 months (IIQ 15 – 58,7 months). Fifty (86.2%) patients were evaluated for the severity of RP, of whom 11(19.0%) experienced bleeding warranting blood transfusion. APC was performed in 38(65.5%) patients diagnosed with RP, and 44.8% of them underwent only one session. A median of one APC session was performed, with a mean of 1.56 sessions. APC outcomes were evaluated by the absence of macroscopic bleeding after sessions, which was achieved in 22(57.9%) patients. This assessment was not possible in three patients (7.9%) owing to the lack of recorded data (Table 3).

**Table 3:** Chronic RP characteristics and treatment outcomes in women with CC undergoing pelvic radiotherapy

Characteristics		n(%)
Time for RP onset*		
	< 6 months	9(15.5)
	Between 6–12 months	28(48.3)
	> 12 months	19(32.7)
	No information	2(3.5)
Clinical RP severity		
	Bleeding on toilet paper or mixed with feces	23(39.6)
	Live blood in the toilet bowl (not mixed with feces)	11(19.0)
	Bleeding requiring blood transfusion	11(19.0)
	Heavy rectal bleeding with clots	5(8.6)
	No information	8(13.8)
Number of APC** sessions		
	None	20(34.5)
	1	26(44.8)
	2–7***	12(20.7)
Macroscopic bleeding after APC		
	No	22(57.9)
	Yes	13(34.2)
	No information	3(7.9)

* RP: radiation proctitis;

** APC: argon plasma coagulation;

*** Intervals between APC sessions ranged from 1–21 months in the patients undergoing this treatment

The presence of RP symptoms according to patient and treatment characteristics revealed a significant association between rectal bleeding and the following variables: brachytherapy (P < 0.001), HTN (P = 0.036) and cumulative radiotherapy dose greater than or equal to 7,000 cGy (P < 0.001) (Table 4).

**Table 4:** Distribution of radiation proctitis (RP) symptoms according to sociodemographic and clinical variables in patients undergoing pelvic radiotherapy

Variables		RP symptoms			p-value*
		Yes	No	Total	
		n(%)	n(%)	n(%)	
Histopathology					0.959
	Adenocarcinoma	9(37.5)	15(62.5)	24(100.0)	
	Squamous cell carcinoma	76(38.8)	120(61.2)	196(100.0)	
	Other histologic results	2(33.3)	4(66.7)	6(100.0)	
Staging					0.082
	I	4(23.5)	13(76.5)	17(100.0)	
	II	43(47.3)	48(52.7)	91(100.0)	
	III	33(37.5)	55(62.5)	88(100.0)	
	IV	7(25.0)	21(75.0)	28(100.0)	
Cancer treatment					0.267
	Radiotherapy + chemotherapy	77(40.7)	112(59.3)	189(100.0)	
	Radiotherapy	4(19.0)	17(81.0)	21(100.0)	
	Radiotherapy + surgery + chemotherapy	2(33.3)	4(66.7)	6(100.0)	
	Radiotherapy + surgery	4(44.4)	5(55.6)	9(100.0)	
Radiotherapy field					0.435
	Pelvis	76(37.6)	126(62.4)	202(100.0)	
	Pelvis + para-aortic	11(45.8)	13(54.2)	24(100.0)	
Brachytherapy					0.001
	Yes	80(44.0)	102(56.0)	182(100.0)	
	No	5(14.3)	30(85.7)	35(100.0)	
Chemotherapy					0.078
	Yes	80(41.0)	115(59.0)	195(100.0)	
	No	6(23.1)	20(76.9)	26(100.0)	
Smoking					0.085
	Yes	20(29.4)	48(70.6)	68(100.0)	
	No	64(41.6)	90(58.4)	154(100.0)	
Diabetes mellitus					0.459
	Yes	10(45.5)	12(54.5)	22(100.0)	
	No	74(37.4)	124(62.6)	198(100.0)	
HTN					0.036
	Yes	37(47.4)	41(52.6)	78(100.0)	
	No	47(33.1)	95(66.9)	142(100.0)	
Nutritional status					0.538
	Malnutrition	5(29.4)	12(70.6)	17(100.0)	
	Normal weight	24(34.3)	46(65.7)	70(100.0)	
	Overweight	26(44.8)	32(55.2)	58(100.0)	
	Obesity	17(40.5)	25(59.5)	42(100.0)	
Cumulative dose of radiotherapy					0.001
	< 7,000	12(17.1)	58(82.9)	70(110.0)	
	≥ 7,000	75(48.1)	81(51.9)	156(100)	

* Pearson's chi-square test

There was no statistically significant association between RP symptoms and the external radiotherapy dose, and no rectal bleeding observed with a dose of up to 4,040 cGy (Table 5).

**Table 5:** Frequency of radiation proctitis (RP) symptoms with progressive doses of pelvic radiotherapy in patients with cervical cancer treated

		RP symptoms		Total n(%)	p-value
		Yes	No		
		n(%)	n(%)		
External radiotherapy dose (cGy)					0.092*
	2,000	0(0.0)	2(100.0)	2(100.0)	
	3,000	0(0.0)	3(100.0)	3(100.0)	
	4,000	0(0.0)	4(100.0)	4(100.0)	
	4,040	0(0.0)	1(100.0)	1(100.0)	
	4,500	6(26.1)	17(73.9)	23(100.0)	
	5,000	58(45.7)	69(54.3)	127(100.0)	
	5,040	23(34.8)	43(65.2)	66(100.0)	

* Fisher's exact test

## Discussion

Herein, we analyzed 226 patients with CC who received pelvic radiotherapy, 58 of whom had RP confirmed colonoscopically. To the best of our knowledge, this study documents the largest number of patients with RP following pelvic radiotherapy administered to treat CC.^(4,23)^

We confirmed the presence of RP in more than 25% of identified patients; this value is higher than that reported previously (20%).^(6,8,23)^ This high incidence could be attributed to most patients in our study receiving a cumulative radiotherapy dose exceeding 8,500 cGy. Cumulative doses exceeding 7,000 cGy were associated with considerable side effects and complications.^(15,23,24)^

RP onset does not depend solely on radiotherapy-related factors. A complex interaction between patient and treatment factors possibly increases RP incidence, severity, and chronicity. The presence of HTN and DM has been associated with an increased risk of RP, especially when these diseases occur concomitantly, which is probably related to microvascular changes in these patients, resulting in impaired repair of tissue damage.^(25,26)^ However, only HTN was significantly associated with rectal bleeding in our study, which could be attributed to the small number of patients with DM.

Rectal bleeding is the most common RP symptom,^(27)^ the clinical marker used in the current study. Patients undergoing colonoscopy showed an almost absolute association between rectal bleeding and RP diagnosis, although one-third of symptomatic patients did not undergo colonoscopy to establish a diagnosis. Therefore, we can suppose that if all patients with rectal bleeding were tested, the percentage of RP in this population could have been higher, approximating 40%.

Given this association between rectal bleeding and RP, the possibility of sparing diagnostic colonoscopy for patients with CC who experience rectal bleeding after pelvic radiotherapy needs to be considered. This would reduce the time to initiate RP treatment, costs, and the number of procedures to prepare for testing.

APC is one of the most widely used treatments, although standardized guidelines regarding the number of sessions needed to control bleeding are lacking, with studies reporting 1–3.7 sessions.^(18)^ Herein, most patients with RP confirmed by colonoscopy underwent no or only one APC session. There was a wide variation in patients who had more than one session, and we could not define the ideal number of sessions and treatment intervals. More than half of the patients achieved bleeding control after APC, even with only one session, corroborating the efficacy of APC reported previously.^(18,28,29)^

We could not gather data regarding hemoglobin levels before and after APC from medical records. Hemoglobin levels are widely used to determine treatment response.^(13,30,31)^ Hence, we employed the absence of macroscopic bleeding to establish APC response. The need for blood transfusion could not be assessed in all patients, given the lack of data in respective medical charts. Thus, the determined percentage of transfusions was well below that reported previously, documented as ~30–50%.^(6)^

Considering the sociodemographic characteristics of the examined sample, it was compatible with characteristics generally attributed to females with CC, as a mean age of 50 years, according to the American Cancer Society.^(32)^ In the current study, almost one-fifth of the patients were >64 years at diagnosis, potentially suggesting a failure to screen this population.

Another factor corroborating the screening failure is that most patients were diagnosed with locally advanced disease, which may reflect the challenges in accessing health services and the consequent low screen coverage in the study region. However, the 2019 epidemiological bulletin on the prevalence of preventive CC testing provided by the Ministry of Health shows that 81.3% of Brazilian females aged 25–64 years reported undergoing CC testing in the previous three years, which may indicate the need to improve the test quality in this population.^(33)^

The histological type found in our study corroborates the type expected for this population, given that squamous cell carcinoma is the most common type, accounting for more than 80% of CC cases in different studies.^(2,34)^ This rate has decreased in recent years, especially in high-income countries, at the expense of an increased incidence of adenocarcinoma, the second most common histological type.^(35)^

Our study was conducted at an oncology referral hospital in a state with a high incidence of CC; hence, it can be considered a representative sample of the analyzed population. The collected data highlights the subject and reinforces the importance of primary and secondary prevention because, in addition to difficulties encountered in treating and curing these patients, a considerable percentage may experience long-term post-treatment complications.

## Conclusion

Our study confirmed the high frequency of RP in patients with CC who underwent pelvic radiotherapy and brachytherapy and demonstrated the association of HTN and high cumulative radiotherapy dose with the onset of RP symptoms.
